# Corrigendum: A combination of metformin and epigallocatechin gallate potentiates glioma chemotherapy *in vivo*

**DOI:** 10.3389/fphar.2023.1322332

**Published:** 2024-01-09

**Authors:** Shreyas S. Kuduvalli, Precilla S. Daisy, Anandraj Vaithy, Mugilarasi Purushothaman, Arumugam Ramachandran Muralidharan, Kumar B. Agiesh, Markus Mezger, Justin S. Antony, Madhu Subramani, Biswajit Dubashi, Indrani Biswas, K. P. Guruprasad, T. S. Anitha

**Affiliations:** ^1^ Mahatma Gandhi Medical Advanced Research Institute (MGMARI), Sri Balaji Vidyapeeth University (Deemed to-be University), Puducherry, India; ^2^ Department of Pathology, Mahatma Gandhi Medical College and Research Institute, Sri Balaji Vidyapeeth University (Deemed to-be University), Puducherry, India; ^3^ Department of Pharmacy, National University of Singapore, Singapore, Singapore; ^4^ Department of Visual Neurosciences, Singapore Eye Research Institute, Singapore, Singapore; ^5^ Eye-APC, Duke-NUS Medical School, Singapore, Singapore; ^6^ Department of General Paediatrics, Haematology/Oncology, University Children’s Hospital Tübingen, Tübingen, Germany; ^7^ ScirosBio (FZC), SRTI Park, Sharjah, United Arab Emirates; ^8^ Department of Medical Oncology, Jawaharlal Institute of Postgraduate Medical Education and Research (JIPMER), Puducherry, India; ^9^ Department of Ageing Research, Manipal School of Life Sciences, MAHE, Manipal, Karnataka, India

**Keywords:** glioma, metformin, epigallocatechin gallate (EGCG), molecular docking, quantum mechanics (QM), ROS-reactive oxygen species, PI3K/Akt/mTOR pathway

In the published article, there was an error in Table 1. The values of drug doses were inadvertently misrepresented during the final assembly of Table 1. The corrected Table 1 and its caption appear below.

**TABLE 1:** The concentration of each drug used in the treatment of animals both individually and in combination.

Groups	Drugs	Dosage (mg/kg body weight)
Group II	Tumor control	NA
Group III	T	50
Group IV	M	80
Group V	E	150
Group VI	TM	50 + 80
Group VII	TE	50 + 150
Group VIII	ME	80 + 150
Group IX	TME	50 + 80 + 150

The authors apologize for this error and state that this does not change the scientific conclusions of the article in any way. The original article has been updated.

