# Supplementary figures and images for: A combination of metformin and epigallocatechin gallate potentiates glioma chemotherapy in vivo
Source: Front Pharmacol. 2023 Mar 21;14:1096614. doi: 10.3389/fphar.2023.1096614 (PMC10070706; doi:10.3389/fphar.2023.1096614)

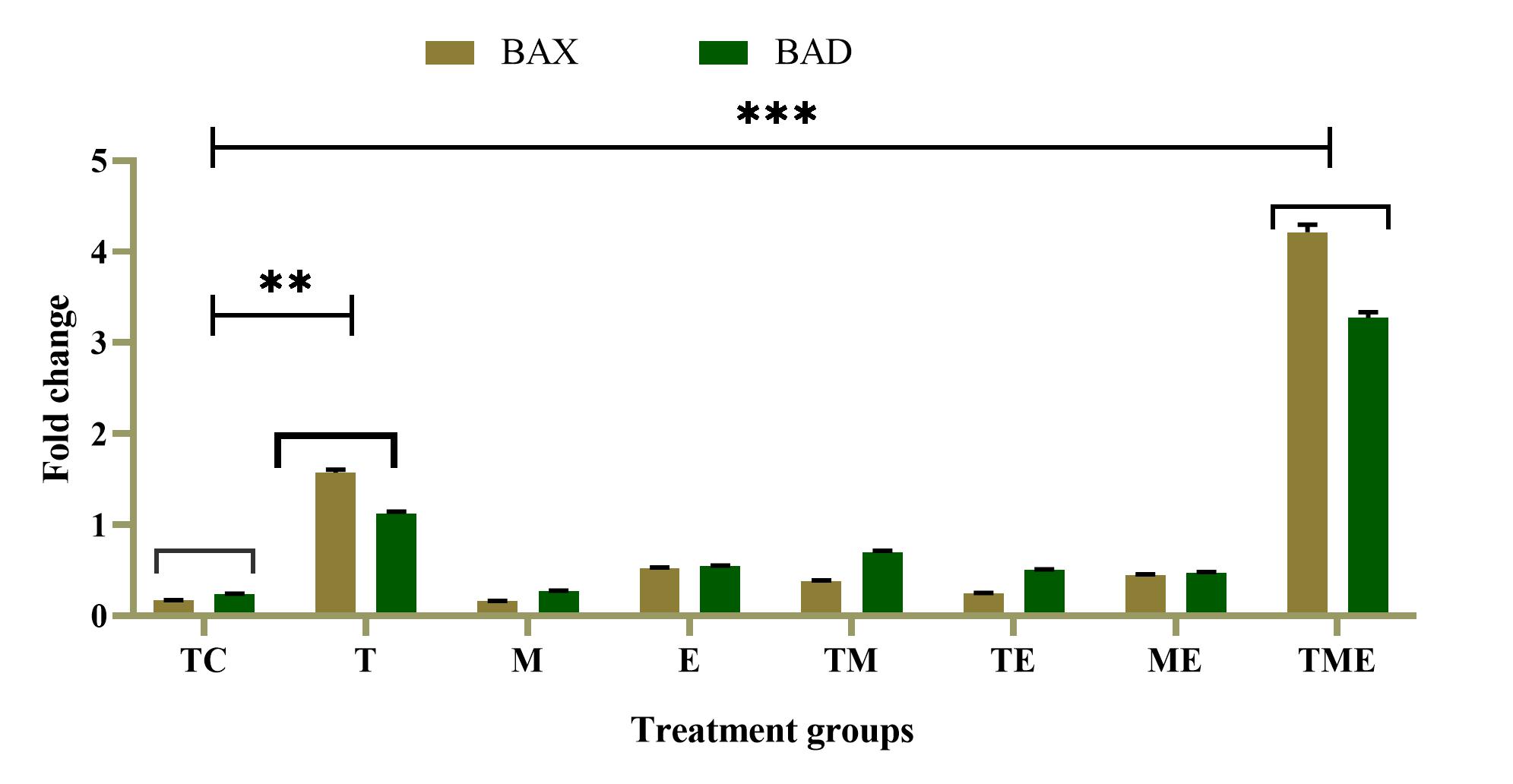

Supplement: Supplementary file 1 [file Image3.JPEG]

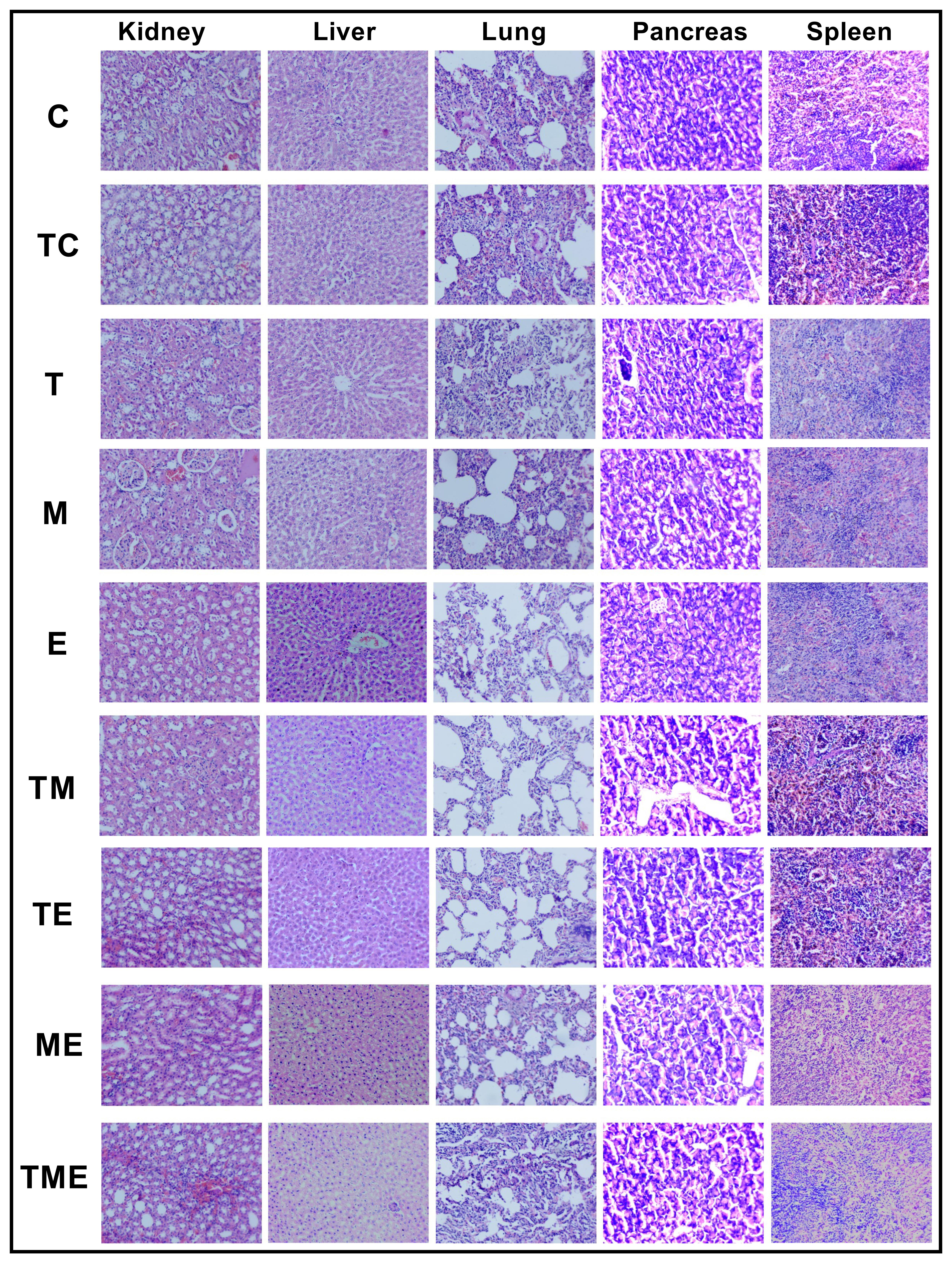

Supplement: Supplementary file 3 [file Image1.JPEG]

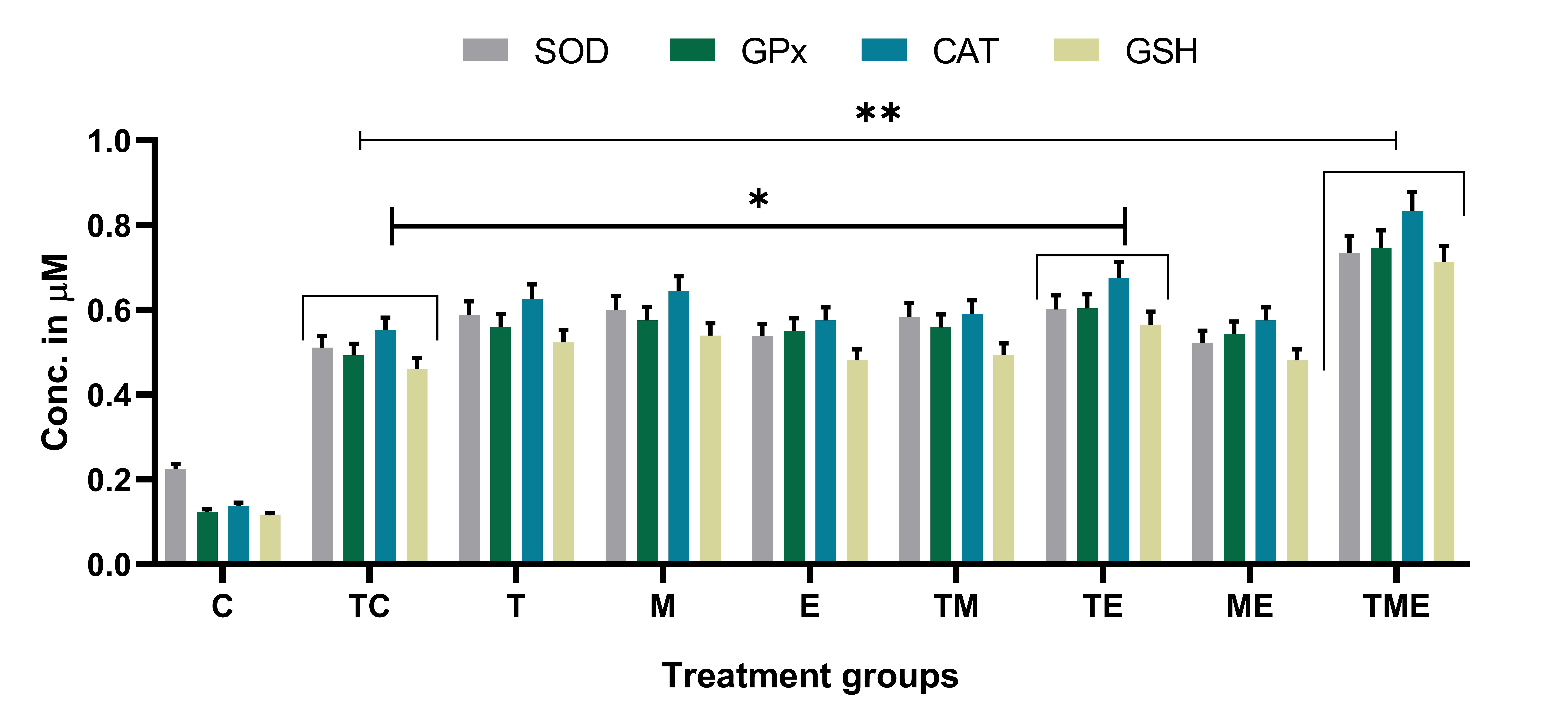

Supplement: Supplementary file 4 [file Image2.JPEG]
