# Supplementary material for: A combination of metformin and epigallocatechin gallate potentiates glioma chemotherapy in vivo
Source: Front Pharmacol. 2023 Mar 21;14:1096614. doi: 10.3389/fphar.2023.1096614 (PMC10070706; doi:10.3389/fphar.2023.1096614)
Supplement: Supplementary file 2 [file Table1.DOCX]

**Supplementary Table S1** Primer sequences used for the qRT-PCR analysis, that were designed using Primer BLAST, NCBI.

| Gene | **Forward (5’-3’)** | **Reverse (5’-3’)** | **ID** |
| --- | --- | --- | --- |
| β-Actin | AGATGACCCAGATCATGTTTGAGA | GCATGAGGGAGCGCGTAA | NM_031144.3 |
| Nrf2 | CAGCATGATGGACTTGGAATTG | GCAAGCGACTCATGGTCATC | NM_031789.2 |
| HIF-1α | GCAACTAGGAACCCGAACCA | TCGACGTTCGGAACTCATCC | NM_024359.2 |
| VEGF | CAAACCTCACCAAAGCCAGC | ACGCGAGTCTGTGTTTTTGC | NM_031836.3 |
| VEGFR-1 (FLT-1) | CAGTGGCTCCACGACCTTAG | GGTGAGGTACGCTGAGCTTT | NM_019306.2 |
| PI3K | TGGCCCGGGTAGGTTTGAAT | ATGCCCTAGGTGACCTGACA | NM_001371300.2 |
| PTEN | AAAGCTGGGAAAGGACGGAC | CACCTTTAGCTGGCAGACCA | NM_031606.1 |
| PDK1 | GGCATAGAGCGGCAGGTTG | AGAAGCGCGCATAGAAGTCC | NM_053826.2 |
| AKT1 | TCATTGAGCGCACCTTCCAT | TTCTGCAGGACACGGTTCTC | NM_033230.3 |
| mTOR | CTGCACTTGTTGTTGCCTCC | ATCTCCCTGGCTGCTCCTTA | NM_019906.2 |
| GSK3-β | AACTCCACCAGAGGCAATCG | AAGCGGCGTTATTGGTCTGT | NM_032080.1 |
| Bax | AGACACCTGAGCTGACCTTGG | GTTGTTGTCCAGTTCATCGCC | NM_017059.2 |
| Bcl2 | GGTGAACTGGGGGAGGATTG | AGAGCGATGTTGTCCACCAG | NM_016993.2 |
| BAD | CTAGGCTTGAGGAAGTCCGAT | CGGGAATGTGGAGCAGATCA | AF279911.1 |
| Caspase-8 | TTTCCATATCAGTCGGCGGG | TCAAGCAGGCTCGAGTTGTC | NM_022277.1 |
| Caspase-9 | AGTTCCCGGGTGCTGTCTAT | GCCATGGTCTTTCTGCTCAC | AF271996.1 |
| Caspase-3 | ATCCACGGAGGTTTCGTTGTTG | TGGGGCCAATAGTGTTTGGTA | NM_012922.2 |
