# Supplementary material for: A combination of metformin and epigallocatechin gallate potentiates glioma chemotherapy in vivo
Source: Front Pharmacol. 2023 Mar 21;14:1096614. doi: 10.3389/fphar.2023.1096614 (PMC10070706; doi:10.3389/fphar.2023.1096614)
Supplement: Supplementary file 5 [file DataSheet1.docx]

**Supplementary figure legends**

**Supplementary Figure S1:** Histopathological analysis of major organs (kidney, liver, lung, pancreas and spleen) of all the experimental groups with H&E stain.

**Supplementary Figure S2:** Shows the levels of antioxidant and non-antioxidant enzymes (SOD, GPx, CAT, GSH) wherein, the triple-drug combination significantly enhanced the levels of all the enzymes followed by the dual-drug treatment (TE) (*P<0.01, ** P<0.001).

**Supplementary Figure S3:** Shows the gene expression levels of pro-apoptotic markers BAX and BAD, wherein, TME significantly elevated the levels of BAX and BAD, followed by the individual treatment with T (**P<0.01, *** P<0.001).
